# Absorption, Metabolic Stability, and Pharmacokinetics of Ginger Phytochemicals

**DOI:** 10.3390/molecules22040553

**Published:** 2017-03-30

**Authors:** Rao Mukkavilli, Chunhua Yang, Reenu Singh Tanwar, Ahmed Ghareeb, Latika Luthra, Ritu Aneja

**Affiliations:** Department of Biology, Georgia State University, Atlanta, GA 30303, USA; vmukkavilli@gsu.edu (R.M.); ychnjcpu@gmail.com (C.Y.); drreenu10@gmail.com (R.S.T.); afghareeb80@gmail.com (A.G.); latika.luthra@yahoo.com (L.L.)

**Keywords:** biopharmaceutical drug disposition classification system, ginger, liquid chromatography tandem mass spectrometry, microsomal stability, pharmacokinetics, solubility

## Abstract

We have previously demonstrated promising anticancer efficacy of orally-fed whole ginger extract (GE) in preclinical prostate models emphasizing the importance of preservation of the natural “milieu”. Essentially, GE primarily includes active ginger phenolics viz., 6-gingerol (6G), 8-gingerol (8G), 10-gingerol (10G), and 6-shogaol (6S). However, the druglikeness properties of active GE phenolics like solubility, stability, and metabolic characteristics are poorly understood. Herein, we determined the physicochemical and biochemical properties of GE phenolics by conducting in vitro assays and mouse pharmacokinetic studies with and without co-administration of ketoconazole (KTZ). GE phenolics showed low to moderate solubility in various pH buffers but were stable in simulated gastric and intestinal fluids, indicating their suitability for oral administration. All GE phenolics were metabolically unstable and showed high intrinsic clearance in mouse, rat, dog, and human liver microsomes. Upon oral administration of 250 mg/kg GE, sub-therapeutic concentrations of GE phenolics were observed. Treatment of plasma samples with β-glucuronidase (βgd) increased the exposure of all GE phenolics by 10 to 700-fold. Co-administration of KTZ with GE increased the exposure of free GE phenolics by 3 to 60-fold. Interestingly, when the same samples were treated with βgd, the exposure of GE phenolics increased by 11 to 60-fold, suggesting inhibition of phase I metabolism by KTZ but little effect on glucuronide conjugation. Correlating the in vitro and in vivo results, it is reasonable to conclude that phase II metabolism seems to be the predominant clearance pathway for GE phenolics. We present evidence that the first-pass metabolism, particularly glucuronide conjugation of GE phenolics, underlies low systemic exposure.

## 1. Introduction

Herbal medicines continue to be the first line of treatment worldwide, with more than 80% of the population consuming them for major and minor illnesses [1]. The ginger root (*Zingiber officinale* Roscoe) has been used as a medicinal herb for more than 2000 years and is one of the most widely consumed dietary supplements in the world [1]. Ginger is used primarily as a remedy for digestive disorders including dyspepsia, colic, nausea, vomiting, gastritis, and diarrhea [2,3]. Studies using animal models have shown that ginger and its phenolic constituents (i.e., 6-gingerol) suppress carcinogenesis in the skin [4,5,6,7,8], gastrointestinal tract [9], colon [10], and breast [11]. Ginger extracts have been tested for both anti-tumor promotion and apoptotic potential in several *invitro* cancer cell lines, including leukemia [12], gastric [13], prostate [14], ovarian [15], and lung carcinoma [16].

The chemopreventive and chemotherapeutic mechanisms of ginger are not well understood but are thought to involve upregulation of carcinogen detoxifying enzymes, anti-oxidants, and anti-inflammatory [17,18,19] activity. Ginger also inhibits nuclear factor κ-light-chain enhancer of activated B cells (NF-κB) activation induced by a variety of agents that have been shown to downregulate NF-κB regulated gene products involved in cellular proliferation and angiogenesis [20], including interleukin-8 (IL-8) [14] and vascular endothelial growth factor (VEGF) [21]. Components of ginger extract (GE) at proapoptotic concentrations induce G_2_/M phase cell cycle arrest and aberrant mitotic cell death associated with tubulin aggregation [22]. It is also reported that GE can induce antioxidant response element (ARE)-reporter gene activity and nuclear factor erythroid 2 related factor 2 (NrF2) expression [23].

While multiple mechanisms have been relatively well-studied on the remedial action of ginger on a spectrum of disease indications, the pharmacokinetic (PK) characteristics of GE phenolics and their correlation with the mechanism of action remains elusive. Only a handful of studies are available that have examined the absorption, bioavailability, metabolism, and elimination of GE phenolics [24,25,26,27]. In particular, only few of the pungent compounds like 6-gingerol (6G), 10-gingerol (10G), 6-shogaol (6S), and zingerone have been investigated [28,29,30,31,32,33]. In addition, reported studies demonstrate administration of 6G using an intravenous bolus route [34,35], which is unlikely to reflect conventional oral dosing for natural medicines. In humans, when ginger was administered up to two grams, and sub-therapeutic concentrations of GE phenolics were detected. Interestingly, glucuronide and sulfate conjugated forms of GE phenolics in plasma have been reported [26].

To better understand and correlate the pharmacodynamic (PD) efficacy with PK characteristics of GE phenolics, we investigated the solubility and stability of GE phenolics in various pH buffers and simulated gastric fluids (SGF) and simulated intestinal fluids (SIF) to assess their suitability for oral dose administration. We assessed their metabolic stability in mouse, rat, dog, and human liver microsomes to gain insights into interspecies differences and their propensity to form phase I metabolites. We performed a mouse pharmacokinetic study with and without co-administration of ketoconazole (KTZ), a known inhibitor of cytochrome P450s and uridine glucuronosyl transferases (UGTs), to assess the effect of first pass metabolism on the exposure of GE phenolics. KTZ is considered a non-specific inhibitor of UGTs with potential effect on UGT1A1, 1A9, 1A6, 1A4, and 2B7 [36,37].

**Figure 1:**
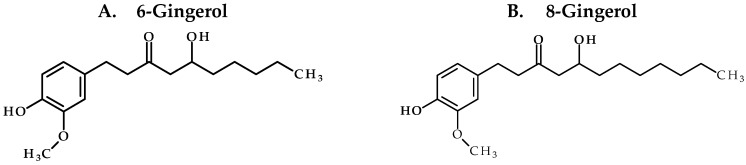

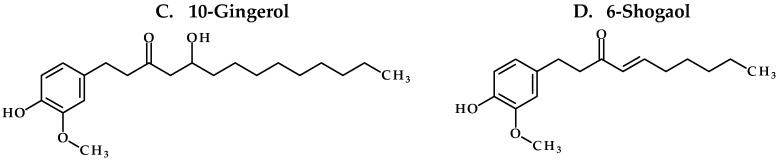
Chemical structures of ginger phenolics.

Our study confirms the suitability of whole ginger extract (GE) for oral dosing as the active GE phenolics (Figure 1) were stable under various pH conditions and in SGF and SIF. However, microsomal stability studies uncovered that GE phenolics undergo extensive phase I metabolism. No species differences were observed, suggesting the appropriateness of animal models for extrapolating the data to humans. The mouse pharmacokinetic study with 250 mg/kg GE showed sub-therapeutic concentration of all GE phenolics. Treatment of plasma samples with β-glucuronidase (βgd) released free GE phenolics, confirming their conversion to glucuronide conjugates. Oral co-administration with 50 mg/kg KTZ did increase the exposure of GE phenolics but did not potently inhibit the phase II glucuronide conjugation reaction. Moreover, the exposure of glucuronide conjugates was far more compared to the increase in exposure due to inhibition of phase I metabolism by KTZ. These results suggest a major role of phase II glucuronide conjugation in clearance of GE phenolics.

## 2. Results

### 2.1. Druglikeness Properties of Ginger Phenolics Based on Structure

GE phenolics followed the Lipinski rule of 5 and Veber rules of druglikeness, confirming their suitability for oral dose administration (Table 1). All GE phenolics had molecular weight of less than 500 daltons and showed polar surface area of less than 140 Å, confirming good permeability.

### 2.2. Biopharmaceutical Classification Based on Solubility

Solubility of GE phenolics was assessed from 10 to 100 µM in hydrochloride, citrate, and phosphate buffers. 6-Gingerol (6G) and 8-gingerol (8G) showed solubility of greater than 100 µM at all tested pH conditions. 10-Gingerol (10G) and 6-shogaol (6S) showed solubility of less than 10 µM under all tested pH conditions (Table 2). Based on the solubility profile of GE phenolics, they may be Class II or Class IV compounds.

### 2.3. Solubility and Stability of Ginger Extract Phenolics in Simulated Gastric and Intestinal Fluids

Solubility and stability of GE phenolics was assessed in SGF and SIF at 10 µM. All GE phenolics were soluble and stable in SGF for 1 h and in SIF for 2 h (Table 3). This confirmed the stability of GE phenolics in the gastrointestinal tract and suitability for oral dose administration.

### 2.4. Liver Microsomal Stability of Ginger Extract Phenolics

**Figure 2:**
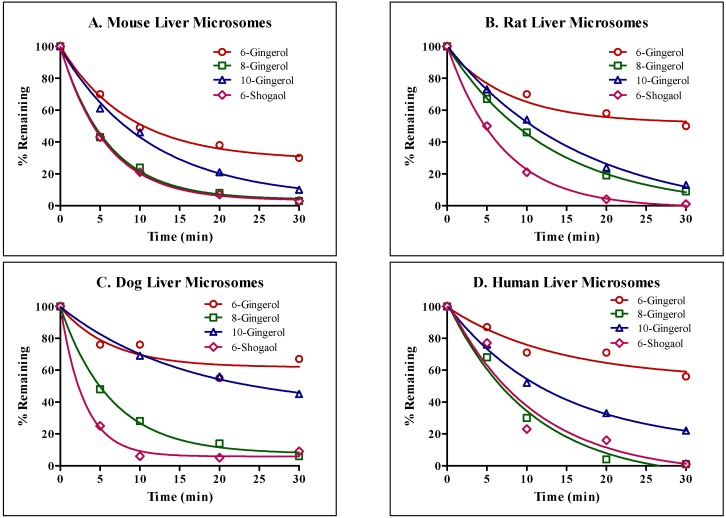
Microsomal stability of GE phenolics. (**A**) Mouse liver microsomes; (**B**) Rat liver microsomes; (**C**) Dog liver microsomes; (**D**) Human liver microsomes.

To assess interspecies differences in metabolic stability of GE phenolics (10 µM), GE phenolics were incubated individually with liver microsomes. Of all the tested GE phenolics, 8G and 6S seemed to be the most unstable, with half-lives of less than 30 min, followed by 6G and 10G. No interspecies difference in microsomal stability of GE phenolics was found, confirming the extrapolation of animal data to humans (Table 4, Figure 2).

### 2.5. Mouse Plasma Stability of Ginger Extract Phenolics

All GE phenolics were individually spiked in mouse plasma at 10 µM and incubated for 60 min at 37 °C. All GE phenolics were found to be stable with half-lives of greater than 60 min. As all GE phenolics were stable in mouse plasma, plasma was considered an appropriate matrix to be collected during pharmacokinetic studies.

### 2.6. Mouse Pharmacokinetic Study

A solution formulation of GE was prepared by using 5% ethanol, 5% Tween 80, 40% polyethylene glycol, and 50% corn oil. The dose volume was 10 mL/kg and the dose administered was 250 mg/kg GE.

**Figure 3:**
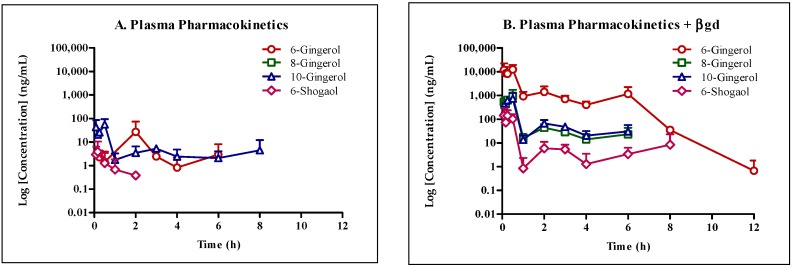
Schematic presentation of concentration-time profile of GE phenolics following oral dose administration of 250 mg/kg GE in mice (*n* = 3 per time point, terminal sampling design, mean ± standard deviation (SD)). (**A**) Plasma pharmacokinetics without β-glucuronidase (βgd) treatment; (**B**) Plasma pharmacokinetics with β-glucuronidase treatment.

Mouse pharmacokinetics study was performed to assess the contribution of phase I and phase II metabolism on the first pass effect of GE phenolics in vivo. To understand the contribution of metabolism on first pass effect, the GE formulation was given with and without co-administration of 50 mg/kg KTZ orally. After collection of plasma samples, one aliquot was processed as such and the other aliquot was treated with βgd for 1 h to deconjugate the glucuronide metabolites. Upon analysis of plasma samples from the GE group, sub-therapeutic concentrations of GE phenolics were observed (Table 5, Figure 3). Following treatment with βgd, the concentration of free GE phenolics increased drastically.

**Figure 4:**
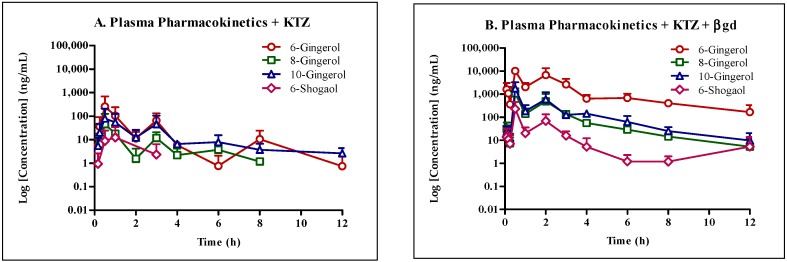
Schematic presentation of concentration-time profile of GE phenolics following oral dose administration of 250 mg/kg GE with 50 mg/kg ketoconazole (KTZ) in mice (*n* = 3 per time point, terminal sampling design, mean ± SD). (**A**) Plasma pharmacokinetics (PK) of GE phenolics with ketoconazole without βgd treatment; (**B**) Plasma PK of GE phenolics with ketoconazole following βgd treatment.

Co-administration of KTZ increased the concentration of free GE phenolics. Treatment of the same samples with βgd showed formation of glucuronide conjugates in vivo (Table 6, Figure 4) suggesting little effect of KTZ in inhibiting the glucuronide conjugation reactions.

The concentration ratio of total (conjugated and free) of GE phenolics to their respective free forms confirmed that phase II conjugation reactions are the most significant and predominant clearance pathways (Table 7).

Comparison of pharmacokinetic parameters of GE dosed alone with and without βgd treatment suggested extensive glucuronidation potential of all studied GE phenolics (Table 8). The increase in area under the curve (AUC_last_) of free GE phenolics in βgd treated samples was 361, 698, 10, and 66-fold higher for 6G, 8G, 10G, and 6S, respectively. Co-administration of KTZ with GE increased the exposure (AUC_last_) of free GE phenolics by 3 to 60-fold, confirming inhibition of phase I and phase II metabolism (Table 8). Further treatment of plasma samples with βgd increased the exposure of free GE phenolics. The increase in exposure was more and within 2-fold compared to GE dosed alone plasma samples treated with βgd confirming little or no effect of KTZ on inhibiting phase II glucuronide conjugation of GE phenolics. Similar trend was observed in maximal plasma concentration (C_max_). Same or earlier time to reach peak plasma concentration (T_max_) further confirmed the propensity of GE phenolics to undergo conjugation reactions and little effect of KTZ on the inhibition of UGTs.

## 3. Discussion

Ginger is being used as a therapy for many clinical conditions with no concrete mechanism of action proven. In human clinical studies, glucuronide and sulfate conjugates of GE phenolics were found in plasma with sub-therapeutic concentrations of free forms following a two-grams dose administered orally. We believe that there is a knowledge gap in tying the efficacy of GE to the analytes responsible for it. To address this gap, we systematically performed various in vitro studies with purified GE phenolics and a pharmacokinetic study in mouse using GE.

We first studied the structural properties of GE phenolics using the Lipinski rule of 5 and Veber rule for druglikeness. All GE phenolics followed the Lipinski and Veber rules confirming their suitability for oral dose administration. Next, we tried to classify GE phenolics according to the biopharmaceutical drug disposition classification system (BDDCS) and assessed their solubility at pH 2, 4, and 7.4. 6G and 8G were found to be soluble up to 100 µM at all three pH conditions. 10G and 6S showed solubility of less than 10 µM. Based on the solubility data and considering a dose of 2 g ginger taken along with 250 mL of water in humans, we classified GE phenolics as Class II or Class IV compounds. To understand the stability of GE phenolics in the gastro-intestinal tract (GIT), we assessed their stability in SGF and SIF. All GE phenolics were found to be stable in SGF and SIF confirming their suitability for oral dose administration. This also confirmed that the sub-therapeutic concentrations of free GE phenolics found in vivo were not due to stability issues in GIT. By using suitable formulation ingredients, solubility of GE phenolics can be improved to achieve desired exposure in vivo. We prepared a solution formulation for GE with various co-solvents for single dose administration.

Next, we compared interspecies differences in phase I metabolism of GE phenolics individually. Liver microsomal stability studies were conducted using mouse, rat, dog and human liver microsomes. All GE phenolics were unstable and showed high intrinsic clearance, with values above 5 mL/min/g, and no interspecies differences in metabolism were observed.

To understand the disposition of GE phenolics in vivo, we conducted a mouse pharmacokinetic study with and without KTZ co-administration and treatment of plasma samples with βgd. The concentration of all GE phenolics increased following treatment with βgd with 6G (361-fold) and 8G (698-fold) showing the highest fold increase in exposure (AUC_last_) followed by 6S (66-fold) and 10G (10-fold). Same or earlier T_max_ of GE phenolics following βgd treatment compared to the non-treated group in the presence or absence of KTZ co-administration confirmed the propensity of GE phenolics to undergo phase II glucuronidation reaction.

Following co-administration with KTZ, the exposure (AUC_last_) of all GE phenolics increased by 3 to 60-fold confirming inhibition of phase I and phase II enzymes. Following treatment of plasma samples with βgd to release free GE phenolics (+KTZ +βgd), the exposure of GE phenolics increased and was within 2-fold of the non KTZ co-administered GE group treated with βgd (−KTZ +βgd, Table 8). In addition, as there was no increase in T_max_ values between the βgd treated and untreated groups, thus it is reasonable to conclude that KTZ did not inhibit the phase II glucuronidation pathway. No significant inhibition seen in the KTZ administered group for glucuronide conjugation could also be due to the lower concentrations achieved in vivo but enough to inhibit phase I biotransformation. This assumption needs to be further confirmed by assessing the concentration of KTZ in vivo and subsequent in vitro phase II glucuronidation inhibition studies in microsomes.

Based on low to moderate solubility and metabolic stability being the major route of elimination and in vivo PK data supporting this, GE phenolics are BCS Class II compounds. To circumvent the sub-therapeutic concentrations seen in vivo, we propose a suitable formulation recipe which can solubilize the GE phenolics to attain desired systemic exposure. Another strategy to increase the systemic exposure of GE phenolics will be to use specific and selective inhibitor(s) for UGTs.

## 4. Materials and Methods

### 4.1. Chemicals

The ginger extract (GE) was a generous gift from Sabinsa Corporation (East Windsor, NJ, USA). The active ginger constituents, 6G, 8G, 10G, and 6S, were isolated from GE by column chromatography and were characterized for >98% purity by high performance liquid chromatography. Acetonitrile (ACN) and methanol were purchased from Fisher Scientific (Pittsburgh, PA, USA). Nicotinamide adenine dinucleotide phosphate (NADPH), Carboxyl methylcellulose (CMC), dihydrocapsaicin, and β-glucuronidase were purchased from Sigma (St. Louis, MO, USA). Mouse, rat, dog, and human liver microsomes were purchased from Xenotech LLC (Kansas City, KS, USA). All other reagents used in the study were of analytical grade.

### 4.2. pH Dependent Solubility of Ginger Extract Phenolics

Kinetic solubility of GE phenolics was assessed from 10 to 100 μM by spiking dimethylsulfoxide (DMSO, Sigma-Aldrich) stock solutions (5 μL, in duplicate) into 995 μL buffer (pH 2.0—hydrochloride, 4.0–100 mM citrate buffer and 7.4–100 mM phosphate buffer) in a 96-well plate and placing at room temperature (22–24 °C) for 2 h. Calibration standards were prepared by spiking 5 μL of DMSO stock solutions into 995 μL acetonitrile/buffer (1:1) mixture. Following centrifugation (10,000 rpm, 10 min, 25 °C) the reaction samples were diluted 1:1 with acetonitrile.

### 4.3. Solubility and Stability in Simulated Gastric Fluid and Intestinal Stability

SGF and SIF were prepared as per USP method. Briefly, SGF was prepared by adding 2 g of sodium chloride and 3.2 g of pepsin with water and pH was adjusted to 1.2 ± 0.1 using hydrochloric acid (7 mL) and volume made up to 1 L. SIF was prepared by dissolving 6.8 g monobasic potassium phosphate and 10 g pancreatin in Milli-Q water (Millipore, Billerica, MA, USA). pH was adjusted to 6.8 ± 0.1 with sodium hydroxide solution and volume was made upto 1 L. Reactions were initiated by spiking DMSO stock solutions of GE phenolics 10 μL into 1990 μL of SGF and SIF incubated at 37 °C for 10 min. Samples (200 μL) were withdrawn at 0, 15, 30, and 60 min for SGF and at 0, 30, 60, and 120 min for SIF and quenched with 200 μL acetonitrile containing internal standard.

### 4.4. Microsomal Stability

Pooled liver microsomes from CD1 mouse, IGS Sprague-Dawley rat, Beagle dog, and mixed gender human were used for assays. Incubations (1 mL) consisted of liver microsomes (0.5 mg/mL), NADPH (1 mM) and 100 mM sodium phosphate buffer (pH 7.4). Following pre-incubation (10 min, 37 °C), reactions were initiated by spiking ACN/DMSO (80:20) stock solutions of GE phenolics (5 μL, 10 μM). Samples (100 μL) were withdrawn at 0, 5, 10, 15, 20 and 30 min and quenched with 200 μL acetonitrile containing internal standard. Concomitant NADPH-free control incubations were prepared similarly.

### 4.5. Plasma Stability Assay

Mouse plasma (995 µL, C57BL/6) was spiked with 5 µL of individual stock solutions of GE phenolics (200 µM stock solution prepared in ACN/DMSO (80:20). After gentle inversion, samples (100 μL) were withdrawn at 0, 15, 30, and 60 min and quenched with 400 μL of ice cold acetonitrile containing internal standard. Assay was performed in duplicate.

### 4.6. Mouse Pharmacokinetic Study

Pharmacokinetic study in male mice (C57BL6J, Harlan Laboratories, Indianapolis, IN, USA) was run with 36 animals in each group at a GE dose of 250 mg/kg with a dose volume of 10 mL/kg. One group was given GE alone and in the second group GE was co-administered with KTZ (50 mg/kg). A terminal sampling design was used to collect blood samples at 0 (pre-dose), 0.08, 0.16, 0.25, 0.5, 1, 2, 3, 4, 6, 8, and 12 h. At each time, 1 mL of blood was collected in ethylenediaminetetraacetic acid di-potassium salt (K_2_EDTA) tubes (BD Biosciences, Franklin Lakes, NJ, USA). Plasma was separated by centrifugation of samples at 4000 rpm for 10 min and stored below −60 °C till bioanalysis. All experiments were performed with approval from the Institutional Animal Care and Use Committee (IACUC, approval code: A14031). All animals were acclimatized for five days before dosing in the experimental area. Food and water was provided ad libitum throughout the study period. Animals were marked and housed (three per cage, *n* = 36 per group) in polypropylene cages and maintained in controlled environmental conditions with 12 h light and dark cycles. The temperature and humidity of the room was maintained between 22 ± 3 °C and 30% to 50%, respectively, and approximately 10–12 fresh air change cycles per hour.

### 4.7. Enzymatic Hydrolysis of Ginger Extract Phenolic Conjugates

To confirm the presence of GE glucuronide conjugates, plasma (100 μL) was treated with βgd (20 μL, 500 units) and incubated at 37 °C for 1 h. Each sample was analyzed twice with and without β-gd treatment. Samples without gde treatment were diluted with 20 μL of buffer. Total (conjugated + free) and free ginger phenolics data were reported following bioanalysis.

### 4.8. Bioanalysis

All in vitro and in vivo samples were processed by protein precipitation method. An aliquot (100 μL) of the sample was spiked with 200 μL of acetonitrile containing dihydrocapsaicin as internal standard (IS) and vortex mixed for 3 min. The tubes were centrifuged at 12,000 rpm for 10 min and an aliquot of supernatant was transferred into autosampler vials for analysis. The stock solutions of 6G, 8G, 10G, and 6S were prepared in ACN/water (70:30) at 1 mg/mL. A calibration curve (CC) range of 2–2000 ng/mL was employed for the quantification of analytes and IS concentration was 150 ng/mL for each analysis. The CC consisted of blank, blank with IS and seven non-zero calibration standards. The calibration standards were within ±15% of the nominal concentration and lower limit of quantification was within ±20% of nominal. All samples were analyzed using liquid chromatography tandem mass spectrometry (LC-MS/MS) method (Agilent 6410 series, Santa Clara, CA, USA). A positive ionization mode with multiple reaction monitoring (MRM, *m*/*z* Q1/Q3) of 6G (*m*/*z* 277.1/177.1, retention time (RT) 2.0 min), 8G (*m*/*z* 305.1/177.1, RT 4.0 min), 10G (*m*/*z* 333.1/177.1, RT 7.7 min), 6S (*m*/*z* 277.1/137.1, RT 5.1 min), and IS (*m*/*z* 308.2/137.1, RT 3.4 min) was employed. The ion spray voltage was set at 3500 V, ionization temperature was set to 300 °C and drying gas flow rate was 10 L/min. Data acquisition and quantitation were performed using Mass Hunter software (Agilent Technologies, Wilmington, DE, USA). Separation was achieved using HP1100 series LC (Agilent Technologies) with an Agilent Zorbax reversed-phase (SB-C18, 2.1 × 50 mm, 5.0 μm) column. A gradient method was employed to separate the individual GE phenolics using mobile phase A (0.1% formic acid in water) and mobile phase B (Acetonitrile). The gradient elution method with 50% B at 0 min, 90% B at 5 min, held for 2.1 min, back to 50% B at 7.1 min with a flow rate of 0.2 mL/min. An injection volume of 2 μL was used for analysis.

### 4.9. Pharmacokinetic Analysis

The pharmacokinetic parameters were calculated from the concentration-time data using the non-compartmental analysis tool of Phoenix WinNonlin software (version 6.3, Pharsight, St. Louis, MO, USA). The area under the concentration-time curve (AUC_last_) was calculated using the linear trapezoidal rule. Following oral administration, peak concentration (C_max_) and time for the peak concentration (T_max_) were the observed values.

### 4.10. Statistical Analysis

Concentration-time profiles were represented as mean ± standard deviation (SD).

## 5. Conclusions

Our studies successfully classified GE phenolics as BDDCS Class II compounds based on solubility and metabolic stability results. We confirmed the suitability of whole ginger extract (GE) for oral dosing as GE phenolics were stable under various pH conditions and in SGF and SIF. No species differences were observed in microsomal stability studies, suggesting the appropriateness of animal models for extrapolating the data to humans. Mouse PK study with GE confirmed the propensity of GE phenolics to form glucuronide conjugates. Co-administration of KTZ with GE decreased the phase I biotranformation but showed little effect on glucuronidation of GE phenolics. The exposure comparison of free GE phenolics and the conjugated forms in the GE alone and GE dosed with KTZ, confirmed affinity of GE phenolics to undergo glucuronide conjugations compared to phase I biotransformation. Our data generates compelling grounds to warrant further studies on the role of phase II conjugates of GE in eliciting the desired efficacy, which may go a long way in the development of ginger as a nutraceutical.

## Figures and Tables

**Table 1:** Druglikeness parameters of ginger phenolics.

Name	6-Gingerol	8-Gingerol	10-Gingerol	6-Shogaol
Polar Surface Area	66.76	66.76	66.76	46.53
Alog P	3.78	4.57	5.36	4.87
Stereo centre count	1	1	1	0
Hydrogen donor	2	2	2	1
Hydrogen acceptor	4	4	4	3
Molecular formula	C_17_H_26_O_4_	C_19_H_30_O_4_	C_21_H_34_O_4_	C_17_H_24_O_3_
Molecular weight	294.38	322.44	350.49	276.37

log P: partition coefficient (octanol-water); Alog P: Atomic log P.

**Table 2:** Solubility of ginger extract (GE) phenolics at various pH conditions.

GE Phenolics	pH 2 ^a^	pH 4 ^b^	pH 7.4 ^c^
6G	>100 µM	>100 µM	>100 µM
8G	>100 µM	>100 µM	>100 µM
10G	<10 µM	<10 µM	<10 µM
6S	<10 µM	<10 µM	<10 µM

^a^ pH 2: hydrochloride buffer; ^b^ pH 4: citrate buffer; ^c^ pH 7.4: phosphate buffer; 6G: 6-gingerol; 8G: 8-gingerol; 10G: 10-gingerol and 6S: 6-shogaol; *n* = 2.

**Table 3:** Stability of GE phenolics in simulated gastric fluid (SGF) and simulated intestinal fluid (SIF).

Experiment	Half-Life (min)			
	6G	8G	10G	6S
SGF (*n* = 2)	>60	>60	>60	>60
SIF (*n* = 2)	>120	>120	>120	>120

Concentration 10 µM; SGF and SIF prepared as per United States Pharmacopeia (USP) method; *n* = 2.

**Table 4:** Liver microsomal stability of GE phenolics.

Liver Microsomes	% Remaining at 30 min				Intrinsic Clearance (mL/min/g Liver)			
	6G	8G	10G	6S	6G	8G	10G	6S
Mouse	30	3	10	3	10.5	15.1	8.0	15.7
Rat	50	9	13	1	11.2	7.0	5.4	13.0
Dog	67	6	45	9	14.1	14.0	5.6	29.9
Human	56	1	22	1	6.6	8.5	6.7	8.4

Substrate concentration: 10 µM; protein concentration: 0.5 mg/mL; slope of one phase exponential decay used for calculating intrinsic clearance = k (min^−1^) × (1/protein concentration in mg/mL) × 45 mg/g; microsomal protein content 45 mg/g liver; *n* = 2.

**Table 5:** Concentration-time profile of GE phenolics following oral dose administration of 250 mg/kg GE in mice.

Time (h)	Concentration (ng/mL)							
	6G		8G		10G		6S	
	−βgd	+βgd	−βgd	+βgd	−βgd	+βgd	−βgd	+βgd
0	0.00 ± 0.00	0.00 ± 0.00	0.00 ± 0.00	0.00 ± 0.00	0.00 ± 0.00	0.00 ± 0.00	0.00 ± 0.00	0.00 ± 0.00
0.087	0.00 ± 0.00	11,818.23 ± 8779.12	0.58 ± 0.45	531.89 ± 176.79	43.07 ± 35.32	204.98 ± 108.13	3.17 ± 2.56	142.97 ± 123.10
0.167	0.11 ± 0.08	8605.37 ± 6006.57	0.20 ± 0.14	438.93 ± 334.64	20.67 ± 6.15	226.51 ± 179.88	4.04 ± 5.37	73.58 ± 93.26
0.25	2.25 ± 1.73	8232.29 ± 2267.36	0.11 ± 0.05	653.31 ± 108.60	25.95 ± 4.43	632.06 ± 44.43	0.39 ± 0.55	141.96 ± 79.78
0.5	1.79 ± 1.82	12,473.13 ± 5596.66	0.23 ± 0.08	948.24 ± 631.34	55.95 ± 31.61	740.17 ± 445.13	1.26 ± 1.78	104.00 ± 46.74
1	1.04 ± 0.79	929.26 ± 397.91	0.42 ± 0.42	16.21 ± 6.22	2.21 ± 0.62	13.74 ± 5.40	0.67 ± 0.95	1.50 ± 0.88
2	27.18 ± 38.43	1428.18 ± 791.01	0.43 ± 0.11	43.94 ± 14.15	4.08 ± 1.61	66.51 ± 20.92	0.38 ± 0.54	6.50 ± 3.47
3	2.45 ± 1.73	711.36 ± 214.33	0.81 ± 0.66	28.68 ± 3.18	5.25 ± 1.06	47.43 ± 6.83	0.00 ± 0.00	5.36 ± 2.50
4	0.82 ± 1.16	402.88 ± 126.92	0.51 ± 0.33	14.05 ± 8.18	3.06 ± 1.19	20.53 ± 9.26	0.00 ± 0.00	2.20 ± 1.21
6	3.22 ± 4.03	1160.60 ± 884.62	0.49 ± 0.30	23.17 ± 16.69	2.43 ± 1.16	30.38 ± 21.34	0.00 ± 0.00	3.89 ± 1.66
8	0.77 ± 0.57	35.30 ± 6.05	0.57 ± 0.23	0.59 ± 0.28	5.04 ± 5.89	1.00 ± 0.34	0.05 ± 0.08	9.31 ± 11.22
12	0.33 ± 0.44	1.07 ± 0.83	0.10 ± 0.05	0.38 ± 0.15	0.62 ± 0.72	0.15 ± 0.05	0.37 ± 0.23	1.76 ± 0.12

**−**βgd: plasma without β-glucuronidase treatment; +βgd: plasma with β-glucuronidase treatment for 1 h at 37 °C; *n* = 3 per time point, terminal sampling design, mean ± SD.

**Table 6:** Concentration-time profile of GE phenolics following oral dose administration of 250 mg/kg GE with 50 mg/kg ketoconazole in mice.

Time (h)	Concentration (ng/mL)							
	6G		8G		10G		6S	
	−βgd	+βgd	−βgd	+βgd	−βgd	+βgd	−βgd	+βgd
0	0.00 ± 0.00	0.00 ± 0.00	0.00 ± 0.00	0.00 ± 0.00	0.00 ± 0.00	0.00 ± 0.00	0.00 ± 0.00	0.00 ± 0.00
0.087	0.31 ± 0.44	1611.34 ± 1136.59	0.48 ± 0.20	31.13 ± 22.39	0.00 ± 0.00	22.80 ± 13.88	0.05 ± 0.07	13.81 ± 15.23
0.167	5.71 ± 4.25	1078.41 ± 788.95	0.63 ± 0.12	25.59 ± 14.92	5.66 ± 5.12	22.03 ± 9.13	0.94 ± 1.33	11.08 ± 8.29
0.25	35.93 ± 44.48	4199.20 ± 5429.93	6.93 ± 9.15	171.88 ± 232.69	20.49 ± 21.65	110.40 ± 138.78	0.00 ± 0.00	22.31 ± 22.45
0.5	37,902.65 ± 53,058.88	35051.01 ± 35340.68	7564.00 ± 10,599.34	5963.41 ± 6931.76	14,015.65 ± 19,653.73	15,312.24 ± 19,191.73	18,637.55 ± 26,338.61	21,051.69 ± 29,448.57
1	103.89 ± 110.27	10,224.71 ± 11,622.13	17.90 ± 19.13	682.37 ± 768.54	54.49 ± 58.26	437.08 ± 369.81	12.46 ± 17.62	170.21 ± 212.35
2	70,928.75 ± 100,284.44	93,896.52 ± 123,421.60	16,340.10 ± 23,104.50	15,805.67 ± 21,666.54	27,873.13 ± 39,392.60	35,370.92 ± 49,184.70	75,903.50 ± 107,343.75	84,223.85 ± 119,013.87
3	65.16 ± 54.26	18,408.37 ± 22,313.19	11.72 ± 7.35	983.51 ± 1199.87	45.84 ± 48.21	603.80 ± 675.86	2.33 ± 3.30	125.05 ± 154.69
4	5.87 ± 1.18	639.17 ± 224.61	2.25 ± 1.56	55.94 ± 15.79	6.52 ± 1.14	142.02 ± 25.61	0.00 ± 0.00	5.17 ± 5.76
6	1.09 ± 0.93	680.17 ± 286.10	3.71 ± 2.18	28.75 ± 14.85	8.07 ± 5.96	62.11 ± 40.75	0.00 ± 0.00	1.20 ± 0.90
8	10.43 ± 11.12	404.60 ± 113.99	1.17 ± 0.11	14.43 ± 2.13	3.71 ± 2.46	25.06 ± 9.52	0.00 ± 0.00	1.19 ± 0.66
12	0.74 ± 1.05	166.32 ± 137.06	0.67 ± 0.17	5.26 ± 5.18	2.61 ± 1.41	9.72 ± 8.83	0.00 ± 0.00	5.27 ± 7.02

**−**βgd: plasma without β-glucuronidase treatment; +βgd: plasma with β-glucuronidase treatment for 1 h at 37 °C; *n* = 3 per time point, terminal sampling design, mean ± SD.

**Table 7:** Concentration ratio of total (conjugated + free) GE phenolics to free forms in GE administered group.

Time (h)	Concentration Ratio (Total/Free) of GE Phenolics			
	6G	8G	10G	6S
0	0	0	0	0
0.087	0	532	5	48
0.167	0	439	11	19
0.25	3659	653	24	0
0.5	8723	948	13	83
1	0	16	8	1
2	53	44	19	16
3	290	29	9	0
4	491	14	9	0
6	391	23	15	0
8	0	0	0	0
12	0	0	0	0

**Table 8:** Pharmacokinetic parameters of GE phenolics with and without ketoconazole co-administration and subsequent treatment of plasma samples with β-glucuronidase.

Compound	Treatment	T_max_ (min)	C_max_ (ng/mL)	AUC_last_ (ng·h/mL)	AUC_last_^a^ Ratio
**6G**	−βgd	120	27.18	37.71	-
	+βgd	30	12,473	13,621	361
	+KTZ −βgd	30	256.7	300.2	8
	+KTZ +βgd	30	10,061	18,782	498, 63 ^b^
**8G**	−βgd	NR ^C^	0.00	0.00	-
	+βgd	30	948.2	697.6	698
	+KTZ −βgd	30	45.68	59.10	59
	+KTZ +βgd	30	1071	1330	1330, 23 ^b^
**10G**	−βgd	30	55.95	61.80	-
	+βgd	30	740.2	634.7	10
	+KTZ −βgd	30	79.00	174.4	3
	+KTZ +βgd	30	1755	1952	32, 11 ^b^
**6S**	−βgd	10	3.880	1.924	-
	+βgd	5	143.0	126.5	66
	+KTZ −βgd	60	12.44	15.08	8
	+KTZ +βgd	30	228.6	212.5	110, 14 ^b^

**−**βgd: plasma samples without β-glucuronidase treatment; +βgd: plasma samples treated with β-glucuronidase; +KTZ **−**βgd: ketoconazole co-administered group plasma samples without β-glucuronidase treatment; +KTZ **+**βgd: ketoconazole co-administered group plasma samples treated with β-glucuronidase; data presented up to 4 significant digits for C_max_ and AUC_last_; ^a^ AUC_last_ ratio calculated considering GE group untreated with βgd as reference; ^b^ AUC_last_ ratio calculated considering GE group co-administered with KTZ untreated with βgd as reference; ^c^ NR: not reportable, C_max_: peak plasma concentration; AUC_last_: Area under the curve; *n* = 3 per time point; mean values reported.

## Abbreviations

The following abbreviations were used in the manuscript:

AUC_last_	Area under the curve
ACN	Acetonitrile
BDDCS	Biopharmaceutical drug disposition classification system
βgd	β-Glucuronidase
CC	Calibration curve
C_max_	Maximal plasma concentration
EDTA	Ethylene diamine tetra acetic acid
GE	Ginger extract
GIT	Gastrointestinal tract
IACUC	Institutional animal care and use committee
IS	Internal standard
KTZ	Ketoconazole
LC-MS/MS	Liquid chromatography tandem mass spectrometry
PD	Pharmacodynamics
PK	Pharmacokinetics
RT	Retention time
SGF	Simulated gastric fluid
SIF	Simulated intestinal fluid
T_max_	Time for peak plasma concentration
UGTs	Uridine glucuronosyl transferases
6G	6-Gingerol
8G	8-Gingerol
10G	10-Gingerol
6S	6-Shogaol
